# Complete Genome Sequences of Four Extensively Drug-Resistant *Pseudomonas aeruginosa* Strains, Isolated from Adults with Ventilator-Associated Pneumonia at a Tertiary Referral Hospital in Mexico City

**DOI:** 10.1128/genomeA.00925-17

**Published:** 2017-09-07

**Authors:** Luis F. Espinosa-Camacho, Gabriela Delgado, Gloria Soberón-Chávez, Luis D. Alcaraz, Jorge Castañon, Rosario Morales-Espinosa

**Affiliations:** a Microbiology and Parasitology Department, Facultad de Medicina, Universidad Nacional Autónoma de México, Mexico City, Mexico; b Molecular Biology and Biotechnology Department, Instituto de Investigaciones Biomédicas, Universidad Nacional Autónoma de México, Mexico City, Mexico; c Laboratorio Nacional de Ciencias de la Sostenibilidad, Instituto de Ecologia, Universidad Nacional Autónoma de México, Mexico City, Mexico; d Intensive Care Unit and Critical Care Unit, Hospital Juarez de Mexico, Mexico City, Mexico

## Abstract

Four extensively drug-resistant *Pseudomonas aeruginosa* strains, isolated from patients with pneumonia, were sequenced using PacBio RS-II single-molecule real-time (SMRT) technology. Genome sequence analysis identified great variability among mobile genetic elements, as well as some previously undescribed genomic islands and new variants of class 1 integrons (In1402, In1403, In1404, and In1408).

## GENOME ANNOUNCEMENT

*Pseudomonas aeruginosa* is a Gram-negative bacterium and is considered to be an opportunistic human pathogen associated with infections in immunocompromised patients. The presence of multidrug-resistant (MDR) and extensively drug-resistant (XDR) strains is associated with high mortality in patients with chronic infections (1, 2). Here, we present the complete genome sequences of four *P. aeruginosa* strains (Pa58, Pa84, Pa124, and Pa127), all isolated from adult patients with pneumonia associated with the use of automatic ventilators in an intensive care unit at a tertiary referral hospital in Mexico City.

Strains Pa58, Pa124, and Pa127 were resistant to different β-lactams, including carbapenems, as well as aminoglycosides and fluoroquinolones. Strain Pa84 was resistant to only imipenem, ciprofloxacin, and levofloxacin. All of the strains were susceptible to polymyxin B. Two strains belong to sequence type 309 (ST-309), a high-risk clone previously documented in Mexico (3).

The genomic DNA of the strains was purified with the DNeasy blood and tissue kit (Qiagen) and sent to the Yale Center for Genome Analysis for PacBio RS-II single-molecule real-time (SMRT) sequencing. A standard library of 20-kb fragments was prepared and sequenced on four SMRT cells with the P4-C2 chemistry. The continuous long reads were assembled using the HGAP/Quiver protocol in SMART portal version 3 (4). The genome features are described in Table 1.

**TABLE 1:** Genome features and accession numbers of four *P. aeruginosa* strains

General composition of genome	Data for strain:			
	Pa58	Pa84	Pa124	Pa127
Sequence type	308	No data	309	309
No. of contigs	2	1	4	13
Coverage (×)	∼83	∼89	∼91	∼91
Genome size (bp)	7,241,575	6,566,724	7,008,516	7,148,302
G+C content (%)	65.80	66.23	65.84	65.74
No. of coding sequences (total)	6,927	6,202	6,617	6,777
No. of tRNAs	63	63	63	63
No. of rRNA operons	4 (5S, 16S, 23S)	4 (5S, 16S, 23S)	4 (5S, 16S, 23S)	4 (5S, 16S, 23S)
No. of noncoding RNAs	4	5	4	4
No. of pseudogenes (total)	244	139	131	205
No. of genomic islands	5	3	3	3
No. of prophages	12	8	10	9
No. of integrative plasmids	3	0	2	2
No. of CRISPR-Cas arrays	1	2	0	0
Class 1 integron(s)	In51, In173, In1212, In1403, and In1404		In1402	In1408
Nucleotide accession no.	CP021775	CP021999	CP021774	CP022000
Biosample accession no.	SAMN05020321	SAMN05020322	SAMN05020323	SAMN05020324

The sequences were annotated using the NCBI Prokaryotic Genome Annotation Pipeline (http://www.ncbi.nlm.nih.gov). The annotation was manually curated, and the genomes were analyzed for the presence of mobile elements (Table 1), antibiotic resistances genes, efflux pumps, and potential virulence factors using the IslandPath-DIMOB, SIGI-HMM, IslandPick (5), CARD (6), VirulenceFinder (7), and Integral (8) databases.

All of the strains have a core genome size averaging 6.15 Mb, with a variable accessory genome. The strains presented different islands inserted at previosly described loci (9). However, these islands present a different gene composition and size than those of the previously reported PAGI-1, PAPI-2, PAGI-4, and pKLC102 strains (10–12). Strains Pa58, Pa124, and Pa127 presented a new island of 70 open reading frames, which is integrated in the tRNA-arg (PA0256) 3′ end. This new island has genes involved in a type I restriction modification system.

Many bacteriophages were detected in all of the strains, most of them having genes involved in phage biogenesis and heavy-metal resistance. Strains Pa58 and Pa84 presented a CRISPR-Cas (clustered regularly interspaced short palindromic repeats and CRISPR-associated genes) array in their chromosomes.

All of the strains showed genes of four efflux pump systems, MexAB, MexCD, MexEF, and MexXY. Strain Pa58 also has genes of the AcrAB efflux pump from *Enterobacter cloacae*. All of the strains carried the OprD, OprJ, and OprM porins, the β-lactams OXA-2 and OXA-50, and the genes *fosA* and *aph*(3′). GyrA presented a mutation at amino acid position 83 (T → I), conferring resistance to fluoroquinolones.

Strains Pa58, Pa124, and Pa127 presented new variants of class 1 integrons, namely, In1402 (http://integrall.bio.ua.pt/?acc=CP021774), In1403 and In1404 (http://integrall.bio.ua.pt/?acc=CP021775), and In1408 (http://integrall.bio.ua.pt/?acc=CP022000), which present the resistance genes *aadA1*, *aadA6*, OXA-2, and *sul1*. All of the strains presented type IV secretion system genes, as well as type III secretion system (*exoU*, *exoS*, *exoT*, and *exoY*) genes.

### Accession number(s).

These whole-genome projects have been deposited in GenBank under the accession numbers listed in Table 1. The BioProject accession number is PRJNA389181.
